# Esophageal Achalasia: Diagnostic Evaluation

**DOI:** 10.1007/s00268-022-06483-3

**Published:** 2022-02-23

**Authors:** Federica Riccio, Mario Costantini, Renato Salvador

**Affiliations:** Department of Surgical, Oncological and Gastroenterological Sciences, School of Medicine, Azienda Ospedale Università di Padova, University of Padova, Padua, Italy

## Abstract

A precise diagnosis is key to the successful treatment of achalasia. Barium swallow, upper endoscopy and high-resolution manometry provide the necessary information about a patient’s anatomy, absence of other diseases, and type of achalasia (I, II, III). High-resolution manometry also has prognostic value, the best results of treatment being obtained in type II achalasia according to the Chicago classification. Abdominal CT scanning and endoscopic ultrasound might be warranted if an underlying malignancy is suspected.

## Introduction

Achalasia is a primary esophageal motility disorder of unclear etiology. It is relatively rare, affecting approximately 1 in 100,000 individuals a year [1]. It is usually diagnosed between 20 and 50 years of age, but can occur at any age, with no predilection for either sex. The disorder is characterized by an impaired lower esophageal sphincter (LES) relaxation and the absence of esophageal peristalsis, resulting in a functional outflow obstruction at the esophagogastric junction (EGJ) [2, 3]. Sir Thomas Willis first described the condition as “cardiospasm” in 1672, and he treated it an event with dilations using a sponge attached to a whale bone. It was not until 1922 that AF Hurst discovered that the motility disorder was due to the LES’s inability to relax, and named it “achalasia” (from the Greek *khalasis*, “relaxation”) [4].

Left untreated, the natural history of achalasia is characterized by a progression towards dilation of the gullet, which gradually becomes more and more enlarged until it acquires an end-stage sigmoid shape. The clinical presentation and symptoms of achalasia include slowly progressing dysphagia for solids and liquids, frequent food regurgitation, or even aspiration (with occasional episodes of pneumonia), chest pain, and weight loss [5]. There is a long delay in many cases between the onset of symptoms and the disorders diagnosis. Patients are often misdiagnosed as cases of heart disease or gastroesophageal reflux disease (GERD) [6]. Reviewing the symptoms of a cohort of patients with diagnosed achalasia, Spechler [7] found that almost half of them reported heartburn. All patients initially suspected of having GERD, but failing to respond to acid suppressant therapy, should be further assessed to exclude esophageal motility disorders such as achalasia. Achalasia patients are also often referred to a psychiatrist for suspected eating disorders, especially if they are young women.

## Diagnostic tests

The diagnosis of esophageal disorders relies basically on three well-established, and often complementary tests: upper endoscopy, barium esophagogram, and (high-resolution) manometry.

### Upper endoscopy

**Fig. 1:**
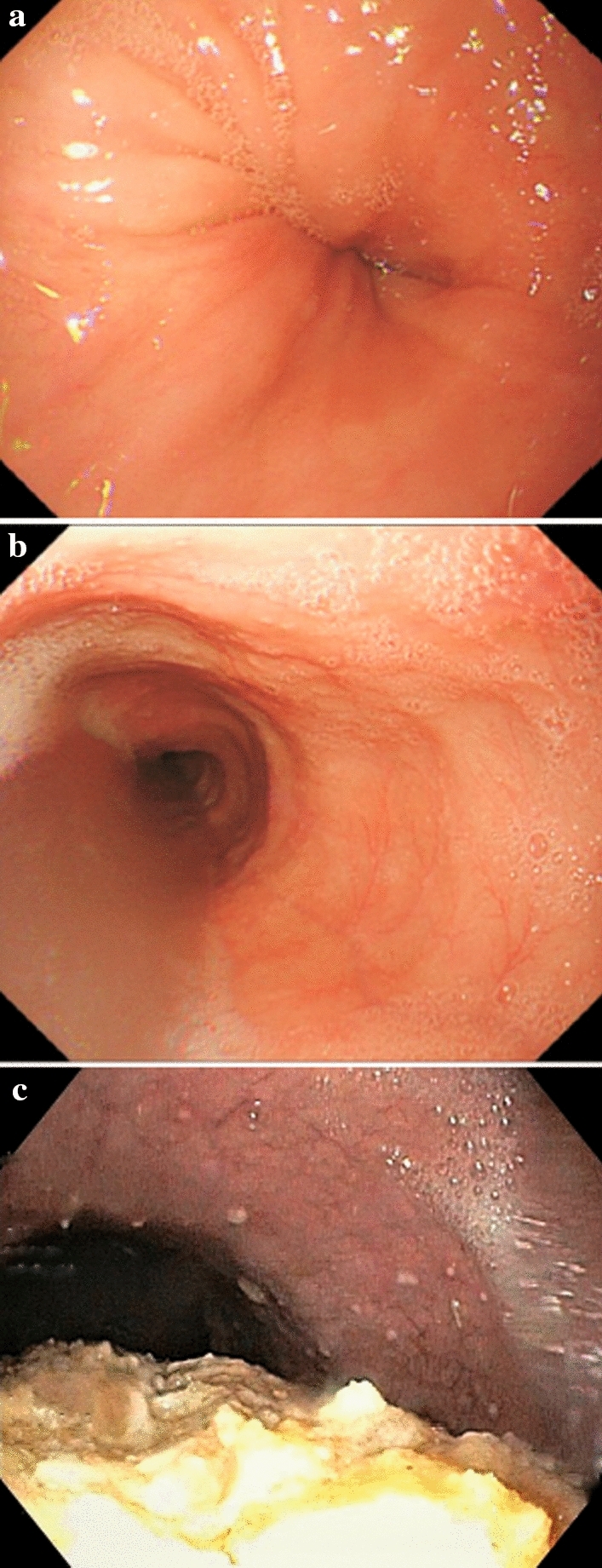
Possible endoscopic findings in patients with achalasia: **a** dilated or tortuous esophagus and tight gastroesophageal junction; **b** dilated esophagus with saliva retention; **c** dilated esophagus with food stasis

All patients referred for dysphagia should first undergo esophagogastroduodenoscopy with mucosal biopsies to exclude other causes of dysphagia, such as erosive GERD, eosinophilic esophagitis, structural lesions (strictures, webs, or rings), and especially esophageal cancer or “pseudoachalasia” [8–10]. Endoscopy is a fundamentally important test, but not very sensitive in establishing a diagnosis of achalasia because more than 40% of patients with achalasia have normal endoscopic findings [11]. That said, evidence of a dilated or tortuous esophagus with saliva and/or food retention, and a tight EGJ on the passage of the endoscope should raise the clinical suspicion of achalasia [10] (Fig. 1).

Esophageal candidiasis refractory to treatment is also common in patients with achalasia, and is caused by esophageal stasis. Finding esophageal candidiasis in patients with an intact immune function should prompt their further assessment for evidence of esophageal dysmotility [10]. This also applies to symptomatic patients with a normal endoscopic picture. During the endoscopy, it is important to perform the retroflexion maneuver in the stomach to rule out any small tumors involving the cardia from below, and causing pseudoachalasia.

### Barium swallow

The aim of barium swallow is to study the capacity for emptying and morphology of the gullet. The test is easy to perform, inexpensive and readily repeatable. The diagnostic sensitivity of barium swallow for achalasia is 60%, while in the remaining 40% of cases the findings are normal or they suggest other disorders [12]. Barium swallow may show an EGJ with the classic “bird’s beak” appearance, a more or less severe dilation of the esophageal body, and a slow passage of the bolus through the junction. A column of retained barium in the esophagus with an air-fluid level is pathognomonic. The test can also reveal some degree of dysmotility up to a gullet with a “corkscrew” appearance, or a complete lack of motor activity, or associated conditions like epiphrenic diverticula, or it can raise the suspicion of an esophageal cancer [13]. The absence of an air bubble in the stomach is a common finding, and strongly suggests a diagnosis of achalasia.

**Fig. 2:**
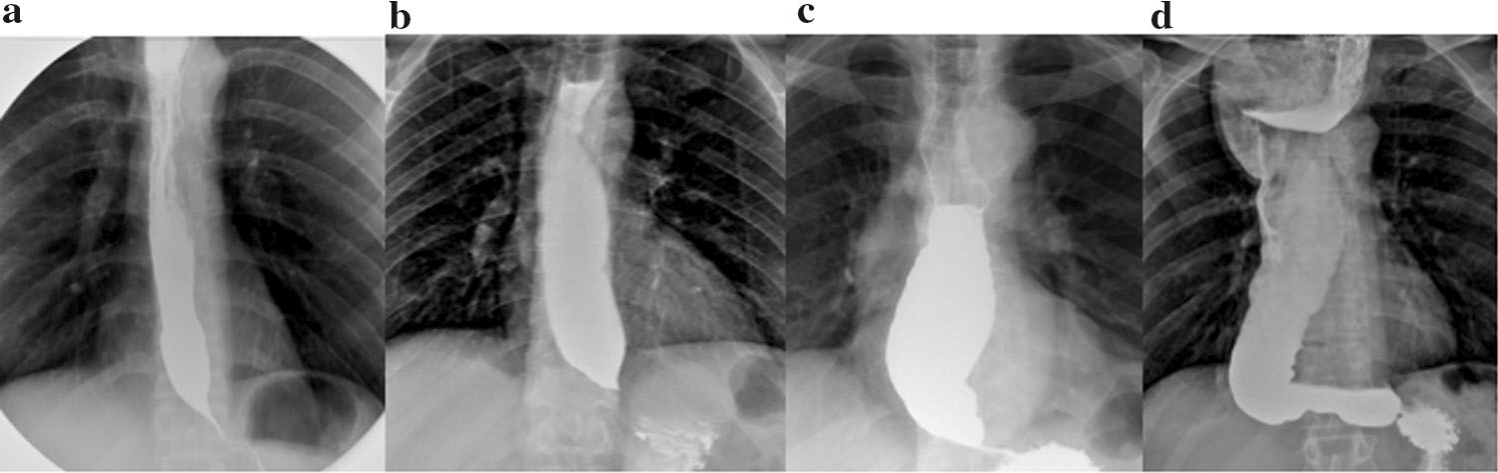
Radiological appearance on barium swallow of different stages of achalasia: **a** stage I, **b** stage II, **c** stage III, **d** stage IV

Different techniques are used to perform a barium swallow. After overnight fasting, patients are asked to swallow a bolus of low-density barium sulfate suspension (45% w/v) while standing. The volume of suspension ingested with every swallow should be as much as they can manage without any regurgitation or aspiration (usually between 100 and 250 ml). The amount of barium ingested should be enough to fill a possibly enlarged esophagus [14]. We can distinguish between four stages of achalasia based on the maximum diameter and shape of the esophagus on barium swallow: stage 1 ≤ 4 cm; stage 2 = 4–6 cm; stage 3 ≥ 6 cm, with a straight esophagus; and stage 4 ≥ 6 cm, with a sigmoid-shaped esophagus (end-stage disease) [15, 16] (Table 1, Fig. 2).

**Table 1:** Radiological stages of achalasia

Radiological stage	Esophageal diameter	Esophageal shape
I	≤ 4 cm	–
II	4−6 cm	–
III	≥ 6 cm	–
IV (End-stage disease)	≥ 6 cm	Sigmoid

A modification of the above-described technique is the “timed” barium swallow. Patients swallow a set amount of barium suspension (200 ml) and images are obtained after 1, 2 and 5 min. This enables the height of the barium column (from its distinct upper level to the EGJ), and the diameter at the widest part of the barium column perpendicular to the long axis of the esophagus to be measured [17]. The degree of esophageal emptying is estimated by comparing the height of the barium on images taken at 1 and 5 min, or by measuring the height and width of each image, roughly calculating the area of the barium-filled esophagus, and assessing the % change in this area in subsequent images. The barium empties from the esophagus completely in 1 min in most healthy controls, and always within 5 min. Esophageal emptying taking more than 5 min suggests achalasia [14, 18, 19]. Timed barium swallows have also proved reliable in the objective assessment of the effects of treatments for achalasia [20]. The cooperation of a dedicated radiologist is essential, however, and may not be readily available at all centers.

**Fig. 3:**
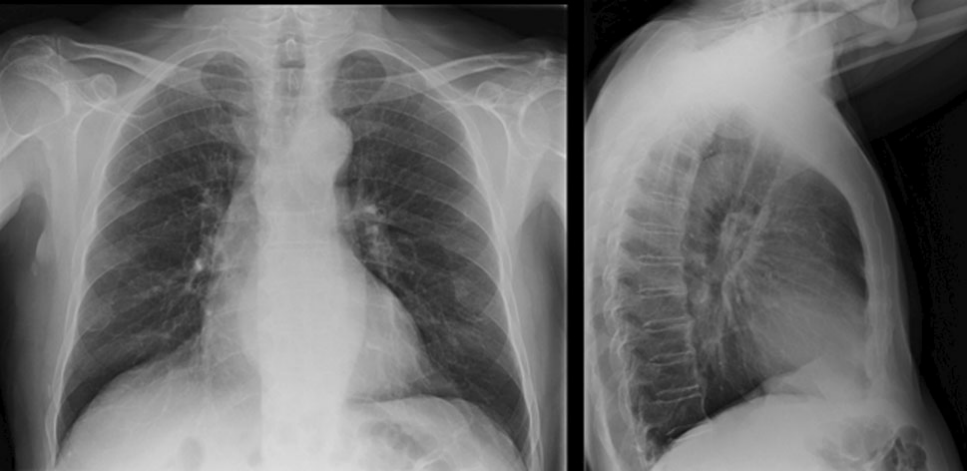
Plain chest X-ray showing a convex opacity overlapping the right mediastinal contour. The gastric bubble is also absent

In patients with longstanding, stage III or IV achalasia, the diagnosis can sometimes be suspected on a simple chest X-ray (in the antero-posterior view) showing a dilated, fluid-filled esophagus (generally seen as a right-sided enlargement of the superior mediastinal profile) and the absence of a gastric bubble [21] (Fig. 3).

### Esophageal manometry

**Fig. 4:**
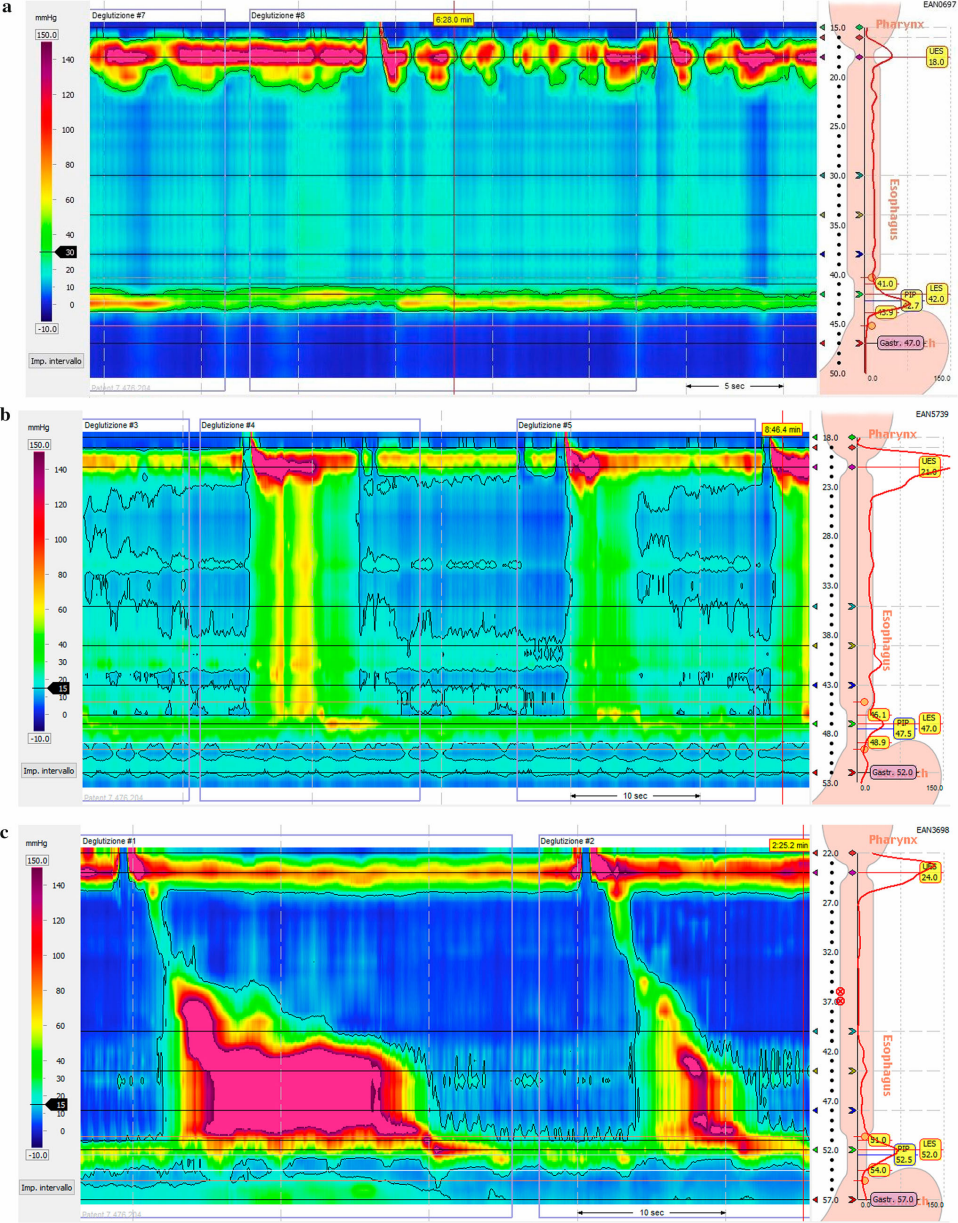
**a** Achalasia Type I. HRM picture showing an impaired EGJ relaxation (IRP > 15 mmHg) with failed peristalsis (distal contractile integral [DCI] < 100 mmHg-s-cm), and without panesophageal pressurization. **b** Achalasia Type II. HRM picture showing an elevated IRP with failed peristalsis and panesophageal pressurization. **c** Achalasia Type III. HRM picture showing an elevated IRP, and a reduced distal latency with a rapidly propagating pressurization and spastic contractions. The first swallow also had a DCI > 5000 mmHg*s*cm

High-resolution esophageal manometry is the gold standard for diagnosing achalasia, based on a lack of peristalsis and an impaired or absent relaxation of the LES in response to swallowing. High-resolution manometry (HRM) of the esophagus only came into clinical practice at the turn of the new millennium, but within just a few years it revolutionized the study of esophageal motility, making the traditional perfused systems obsolete [22]. This led to the development of a new classification of esophageal motility disorders, the Chicago Classification (CC), based right from the start on a dichotomy where impairment of EGJ relaxation marks the great divide among esophageal motility disorders. EGJ relaxation is considered impaired when the integrated relaxation pressure (IRP) measured during a 4-s interval is above 15 mmHg. Then, if esophageal body peristalsis occurs simultaneously or is completely lacking, then achalasia is diagnosed. According to the CC (recently released in its 4th version), [23] achalasia can be divided into 3 subtypes: type I, with an abnormal median IRP and no contractility (100% failed peristalsis); type II, with an abnormal median IRP, no contractility (100% failed peristalsis), but more than 20% of swallows with panesophageal pressurization; and type III, with an abnormal median IRP, more than 20% of swallows with premature or spastic contractions, and no evidence of peristalsis [23]. (Fig. 4 a–c) It is very important to identify the subtype of achalasia in a given patient because there is clear evidence of it serving as an independent predictor of the success of the various treatments for the disorder [24, 25]. It is still not clear whether the three types of achalasia envisaged by the CC are different phenotypes of the disease or represent different stages in its evolution. Evidence has recently emerged that strongly supports the latter hypothesis, where type III would be an early stage, type II an intermediate stage, and type I the end stage of achalasia. Some cases of transition from one type to another, or from a different motor disorder (distal esophageal spasm, EGJ outflow obstruction) to achalasia have also been described, further supporting a hypothesis labeled as the “Padova Theory” (from the group that first suggested it) [26].

## Other tests

### Functional lumen imaging probe

The functional lumen imaging probe (FLIP) is a new technology that delivers real-time, simultaneous measurements of the pressure and diameter of the esophagus in a simulated 3D model. The FLIP uses high-resolution impedance planimetry to measure multiple adjacent cross-sectional areas (CSAs), as detected by a cylindrical bag placed on the catheter during volume-controlled distensions. Measuring the intra-bag pressure during a distension enables an assessment of the CSA-pressure relationship (or distensibility) of the area involved. The distensibility of the EGJ is assessed from its distensibility index, measured as the narrowest diameter corresponding to the greatest distending pressure. This index is abnormally low in untreated achalasia patients [27]. FLIP topography may also identify propagating contractions in achalasia patients with no peristalsis on HRM [28]. Rohof et al. showed that EGJ distensibility can predict the efficacy of treatment for achalasia better than HRM and LES pressure [29]. More data are needed, however, before this expensive test can be used in the clinical diagnosis of achalasia, or for assessing the efficacy of its treatment.

### CT scanning

Pseudoachalasia (or secondary achalasia) is an achalasia-like motility disorder that can be caused by various esophageal and extraesophageal conditions (such as small tumors of the cardia, peptic strictures, the sequelae of surgery). Unfortunately, barium swallow—and even HRM—can rarely differentiate between primary achalasia and pseudoachalasia. CT scanning can have an important role in this context, however, because esophageal narrowing is a common finding on CT scans in both primary achalasia and pseudoachalasia, but the narrowed segment tends to be smooth in patients with the former and uneven in those with the latter. Distal wall thickening tends to be nodular/lobulate and asymmetric in patients with pseudoachalasia. CT scanning should therefore be recommended in patients > 50 years old with a recent and rapidly-evolving history of dysphagia. Any mediastinal lymphadenopathy is also relatively specific for pseudoachalasia, and so are distant metastases, although patients with malignancies in the chest or elsewhere may occasionally have primary achalasia too [30].

### Endoscopic ultrasonography

Another useful test for distinguishing between primary achalasia and pseudoachalasia is endoscopic ultrasonography. In the early stages, cancers of the EGJ may grow within the esophageal wall, leaving the mucosa intact. As this condition can be confused with esophageal achalasia on standard endoscopy, endoscopic ultrasonography could be very useful in shedding light on such cases [31].
